# How Soon Do Depression and Anxiety Symptoms Improve after Bariatric Surgery?

**DOI:** 10.3390/healthcare11060862

**Published:** 2023-03-15

**Authors:** Laura Aylward, Christa Lilly, Madeline Konsor, Stephanie Cox, Salim Abunnaja, Nova Szoka, Lawrence Tabone

**Affiliations:** 1 Department of Behavioral Medicine and Psychiatry, West Virginia University School of Medicine, Morgantown, WV 26506, USA; 2 Department of Epidemiology and Biostatistics, West Virginia University School of Medicine, Morgantown, WV 26506, USA; 3 Department of Psychiatry & Behavioral Sciences, Rush University Medical Center, Chicago, IL 60612, USA; 4 Department of Surgery, West Virginia University School of Medicine, Morgantown, WV 26506, USA

**Keywords:** PROMIS, course, mental health, weight loss, United States, postoperative, intervention, mood, affective, self-efficacy

## Abstract

Depression and anxiety are prevalent among bariatric surgery candidates, yet little is known about the course of symptoms after surgery. This study aimed to identify how soon changes in depression and anxiety occur after surgery. A retrospective review of patients treated at a university hospital was conducted. Participants attended a presurgical psychological evaluation, completed surgery, and attended follow-up visits with bariatric medical providers (2 weeks, 6 weeks, 3 months, and 6 months postoperatively). Depression and anxiety symptoms were assessed at all time points by the Patient-Reported Outcomes Measurement Information System (PROMIS) Depression and Anxiety. Generalized estimating equations models with repeated measures by person over time were used to examine change in depression and anxiety symptoms across time. Among 27 patients, anxiety (incident rate ratio (IRR) = 0.81, *p* = 0.04) and depression (IRR = 0.78, *p* = 0.05) significantly improved both 6 weeks and 3–6 months after bariatric surgery, after controlling for education, marital status, surgery type, age, and baseline body mass index. This is the first known study to show faster improvement in anxiety compared to depression after bariatric surgery. Understanding reductions in anxiety and depression symptoms may be important for postoperative care and timing of weight maintenance interventions.

## 1. Introduction

Depression and anxiety are common comorbid concerns associated with being overweight or obese [1,2]. For those seeking bariatric surgery, it is best practice for affective symptoms to be assessed during the presurgical psychological evaluation [3]. Assessment of depression and anxiety symptoms is important because both uncontrolled and controlled affective symptoms have been associated with negative short- and long-term postsurgical outcomes [3,4,5,6]. Less research has been conducted on psychological factors post-surgically, therefore much less is known about the course of anxiety and depression symptoms after bariatric surgery.

Depression has been shown to improve after bariatric surgery. In a sample of low-income patients who completed the Beck Depression Inventory (BDI) before and after surgery, depression significantly improved at 6 months post-surgery compared to baseline, and this effect continued 12 months post-surgery [4]. Similar results have been seen in other studies [5,6,7,8]. Effect size was reported in one study and demonstrated that improvement in depression from pre- to post-surgery had a large effect size [6]. A recent systematic review and meta-analysis revealed that depression improved following bariatric surgery regardless of surgery type, assessment tool for depression, and follow-up status [5]. In this review, follow-up time varied; however, the median was 24 months. When subgroup analyses were conducted at 6, 12, 24, 36, 48, and 60 months after surgery, depression was significantly lower at every time point compared to prior to surgery [5]. This indicated that the course of depressive symptoms appeared to be independent of specific surgery factors and warrants further investigation. Beyond the assessment of depressive symptoms, the prevalence of depressive disorders has also been shown to decrease after bariatric surgery [7].

Compared to depression, anxiety is a more prevalent psychiatric concern among bariatric surgery patients at the time of the psychological evaluation [2,9]. However, less is known about the course of anxiety after bariatric surgery [10]. In a systematic review that examined outcomes at and beyond 24 months after bariatric surgery [11], authors concluded that overall reductions in anxiety occurred ≥24 months afterwards. However, upon review of the results, the course of anxiety as an independent construct was difficult to determine. Of the eight studies the review included, at least four assessed anxiety through the Hospital Anxiety and Depression Scale (HADS) and only reported the total score, which also included a measurement of depression symptoms. The authors acknowledged that anxiety outcomes are less understood due to a lack of studies that examine anxiety separately from depression [11].

It has also been hypothesized that anxiety tends to be less impacted by bariatric surgery, and therefore the course remains similar before and after surgery [7,10]. For example, one study that assessed anxiety disorders (i.e., panic disorder, agoraphobia, social phobia, specific phobia, obsessive compulsive disorder, post-traumatic stress disorder, and generalized anxiety disorder) via the Structured Clinical Interview for DSM Disorders (Diagnostic and Statistical Manual of Mental Disorders) found no significant change in prevalence of anxiety disorders two years after bariatric surgery [7]. They also found that bariatric patients with diagnosed anxiety and depression prior to surgery lost significantly less weight [7]. This study differs from most, as it focused only on diagnosed mental health disorders rather than symptoms. Another study examined the course of anxiety, as measured by the HADS anxiety subscale, prior to surgery and 1, 2, and 3 years postoperatively [12]. Anxiety significantly decreased from before surgery to 1 year afterwards; however, given that the mean difference was one point, it is unclear if this study held clinically relevant meaning. Additionally, anxiety was not significantly different at baseline compared to 2 or 3 years after surgery [12]. Authors proposed that anxiety may be “a more weight-independent trait pattern” [12]. Currently, findings from studies evaluating the prevalence and course of anxiety symptoms after bariatric surgery are mixed.

While some studies have looked at the course of depression and anxiety after bariatric surgery, studies have not asked how soon potential changes are observed. Most available data that demonstrates improvements in depression are reported around 6 months postoperatively. For example, one study showed that scores on the BDI significantly decreased from baseline to 20 weeks [13]. Given that depression and anxiety are rarely the primary outcome variables of bariatric studies [10,11], it is even less likely that studies are evaluating the timing of these potential affective changes. Current studies have primarily examined longer-term changes (i.e., >6 months); therefore, it is unknown if improvements are observed sooner.

The aim of this study was to identify how soon after bariatric surgery changes in depression and anxiety symptoms occur. Because health behavior engagement can be adversely impacted by depression and anxiety symptoms [14,15,16,17,18,19], changes in affective symptoms may present opportunities to intervene and improve surgical outcomes [3]. More specifically, a reduction in depression symptoms may increase adherence and behavioral activation related to postoperative care. Relatedly, a reduction in anxiety symptoms may also contribute to greater feelings of self-efficacy and positive coping with postoperative body changes [3,14]. Identifying the timing of changes may provide opportunities for impactful change at postoperative visits.

## 2. Materials and Methods

### 2.1. Participants

Study participants consisted of patients who underwent bariatric surgery and completed postoperative clinic visits. All data were collected as part of routine clinical care. Patients were eligible for surgery if they aged ≥18 years and presented with body mass index (BMI) ≥ 40 kg/m^2^ or BMI ≥ 35 kg/m^2^ with co-morbid medical conditions such as obstructive sleep apnea, type 2 diabetes, and/or hypertension.

### 2.2. Measures

#### 2.2.1. Demographics

Participants’ age, sex, race, educational attainment, employment status, and marital status were collected during the pre-surgical psychological evaluation (baseline).

#### 2.2.2. Depression and Anxiety

The National Institutes of Health (NIH) Patient-Reported Outcomes Measurement Information System (PROMIS) has short forms to assess depression and anxiety. Both are publicly available and written for an average reading level of first grade [20]. The PROMIS Depression Short Form (PROMIS-D; formally called LEVEL 2—Depression—Adult PROMIS Emotional Distress—Depression—Short Form) contains 8 self-report questions. Responses to the 8 items are summed to produce a raw total score. The questions ask respondents about the past seven days and how often the symptoms bothered them (i.e., never, rarely, sometimes, often, or always). Some items are “I felt worthless”, “I felt sad”, and “I felt hopeless.”

The PROMIS Anxiety Short Form (PROMIS-A; formally called LEVEL 2—Anxiety—Adult PROMIS Emotional Distress—Anxiety—Short Form) contains 7 self-report questions, and scoring follows the same methods as for the PROMIS D. These questions also ask respondents about the past seven days and how often the symptoms bothered (i.e., never, rarely, sometimes, often, or always). Some items are “I felt anxious” and “I found it hard to focus on anything other than my anxiety”.

For both questionnaires, higher scores indicate higher levels of said construct. In this study, depression and anxiety symptoms were assessed at all time points (baseline, 2 weeks, 6 weeks, 3 months, and 6 months postoperatively). Psychometric properties of both the PROMIS-D and PROMIS-A were previously examined among a sample of bariatric surgery candidates, and they showed good reliability, validity, and invariance [21]. In the present study, Cronbach’s alpha ranged from 0.84 to 0.96 for PROMIS-D and 0.88 to 0.93 for PROMIS-A.

#### 2.2.3. Body Mass Index

Data for objective BMI (participant height and weight) were extracted from the patient’s electronic medical record.

### 2.3. Analysis

All analyses were conducted in SAS 9.4 (SAS Institute, Cary, NC, USA). Descriptive statistics included means and standard deviations for continuous variables and frequencies and valid percentages for categorical variables. Continuous variables were generally positively skewed. Generalized estimating equation (GEE) models were run with negative binomial distributions, log links, and repeated measures by person over time for all outcomes; estimates were exponentiated and interpreted as incident rate ratios. Some covariates (i.e., education, surgery type, marital status) were dichotomized to increase cell size count. Time 0 is baseline, Time 1 is 2 weeks post-surgery, Time 2 is 6 weeks post-surgery, and Time 3 is 3–6 months post-surgery.

## 3. Results

### 3.1. Descriptive Statistics

Demographic and descriptive variables are presented in Table 1. The majority of participants identified as female (88.9%) and average age was 40.4 years. All participants identified as Caucasian, and a majority were married (55.6%). Modal education level was some college and nearly all identified their work status as full-time (92.6%). Most patients underwent laparoscopic sleeve gastrectomy (76.9%).

At baseline, average BMI was 48.13 kg/m^2^ (considered class III or “severe” obesity), PROMIS-D was 13.24, and PROMIS-A was 12.52. See Table 1 for descriptive statistics of outcome variables per time point.

### 3.2. Inferential Statistics

#### 3.2.1. BMI

BMI steadily decreased from M = 48.13 at baseline to M = 36.07 at time 3 (Table 1). This was reflected in the GEE model (Table 2), where baseline scores significantly differed from Time 1 (IRR = 0.92, *p* < 0.0001), Time 2 (IRR = 0.90, *p* < 0.0001), and Time 3 (IRR = 0.79, *p* < 0.0001), even after controlling for covariates of education, marital status, surgery type, and age in years.

#### 3.2.2. PROMIS-D

PROMIS-D decreased from M = 12.5 at baseline to M = 10.7 at Time 1, increased slightly to M = 11.3 at Time 2, and then decreased again to M = 9.8 at Time 3 (Table 2). The GEE model (Table 2) showed baseline scores significantly differed from Time 3 (IRR = 0.78, *p* = 0.05), although the decrease at Time 1 nears statistical significance (IRR = 0.83, *p* = 0.08), even after controlling for covariates of education, marital status, surgery type, age in years, and baseline BMI.

#### 3.2.3. PROMIS-A

PROMIS-A decreased from M = 13.2 at baseline to M = 10.8 at Time 1 (Table 1), and then remained fairly stable at Time 2 (M = 10.9) and Time 3 (M = 11.4). The GEE model (Table 2) demonstrated that baseline scores significantly differed from Time 2 (IRR = 0.81, *p* = 0.04), even after controlling for covariates of education, marital status, surgery type, age in years, and baseline BMI. No significant change was observed between baseline scores and Time 1 (IRR = 0.80, *p* = 0.07).

#### 3.2.4. Clinical Interpretation

Depression and anxiety were reported as raw scores. To aid in interpretation, raw scores were converted to standardized t scores (with a mean of 50 and standard deviation of 10), averaged, and interpreted according to guidelines of minimal important change [22] (see Table 3). The change in t-scores was calculated and interpreted in the context of the standards for minimally important differences (MID) [23]. There are various guidelines for what is considered MID or minimal important change (MIC). Terwee et al. state that a change of between 2 and 6 t-score points is considered a minimal important change [24]. However, a change of 3 t-score points was considered reasonable to PROMIS leadership [22]. In a study that used the PROMIS emotional well-being depression and anxiety short form specifically, a change of 2.3–3.4 t-score points for anxiety and 3.0–3.1 t-score points for depression were considered minimally important differences [25].

The change in t-score for depression was 4.79. The change in t-score for anxiety was 5.44. Regardless of the guidelines used, the results of the present study are considered clinically meaningful changes for both depression and anxiety symptoms.

## 4. Discussion

Anxiety and depression symptoms significantly improved both 6 weeks and 3–6 months after bariatric surgery. This is the first known study to show faster improvement in symptoms of anxiety compared to depression following bariatric surgery. Improvement in symptoms was independent of preoperative BMI. Examining anxiety and depression independently and as outcomes following bariatric surgery is relatively novel. Postoperative follow-up visits with a mental health provider are not standard across bariatric surgery programs, meaning depression and anxiety symptoms are more easily overlooked. Additionally, general postoperative follow-up rates are low, ranging from 3–50% depending on the study or clinic [26]. Both factors present barriers to research on affective symptoms postoperatively.

Findings showed that not only did anxiety symptoms improve after bariatric surgery, but also that this improvement occurred faster than the improvement in depression symptoms following surgery. Given the dearth literature on anxiety symptoms after surgery, there are limited previous results to compare with the present results. The only other study we are aware of that demonstrated a significant change in anxiety after surgery occurred one year after [12]. Some research suggests that anxiety is relatively consistent over time and would be unlikely to change after bariatric surgery [10]. While that may be true for a formally diagnosed anxiety disorder, the present study refutes speculation that individual symptoms of anxiety go unchanged. Due to demonstrated improvement in worry and associated symptoms, 6 weeks after surgery may present a unique opportunity to perpetuate positive health behavior changes at a post-surgical follow-up appointment. Self-efficacy is impacted by the surgical recovery process [14] and has been shown to improve one year after surgery for those who previously had low self-efficacy [27]. Early postoperative appointments are an influential time in patients’ lifelong health changes, and decreased anxiety may enhance patients’ ability to meaningfully engage in follow-up appointments.

Another possible explanation for the results is that patients may be anxious about the procedure itself and they experience a reduction in anxiety symptoms after the perioperative period. The bariatric clinic in the current study has standard follow-up visits with patients at 2 weeks, 6 weeks, 3 months, 6 months, and 12 months postoperatively. It is likely that patients who receive a positive report from their medical providers at 6 weeks may experience a reduction in anxiety symptoms, such as worry or fearfulness. Relatedly, many patients are often concerned about the transition from a liquid diet to solid foods. Improvement or remission of potential complications, such as vomiting, pain, dumping syndrome, food intolerances, and nausea may have occurred by 6 weeks postoperatively, therefore reducing their anxiety about the transition.

Improvements in depression were also observed in the present study, though at a later point postoperatively. Our result that depression improved after surgery is consistent with previous studies; however, improvements were observed sooner after surgery compared to other studies. The earliest that studies typically found improvements in depression was at 6 months [4,7]. Patients pursuing bariatric surgery are often motivated towards weight loss due to improved management of comorbid health conditions, longevity, and/or increased quality of life [3]. It is plausible that patients who are further out from surgery feel more hopeful about their future, which in turn positively impacts symptoms of depression, including hopelessness and disinterest in activities.

Similar to other studied weight-related comorbidities, improvement in affective symptoms may be independent of weight loss after metabolic surgery and have greater response to biochemical changes from surgery. Given that the brain is an organ with high metabolic and nutrient demands, it is probable that bariatric surgery contributes to improvements in affective symptoms independent of weight loss. Nearly 30 years ago, Pories et al. published a pivotal article demonstrating the rapid normalization of blood glucose levels in patients with type 2 diabetes mellitus after bariatric surgery [28]. Several other hormonal changes following bariatric surgery have been found, such as a reduction in obesity-induced corticotropic axis activation and improvements in gonadal profile and insulin resistance. This favorable metabolic profile after surgery is associated with reduction of all-cause mortality [29,30,31] as well as a reduction of comorbidities such as osteoarthritis, respiratory dysfunction, polycystic ovary syndrome, and resolution of cardiovascular risk factors [32,33,34].

One might hypothesize that the dietary education received, vitamin supplementation, and healthy diet following surgery plays an important role in the early observed improvement in mental health. Patients with obesity often deal with maladaptive eating patterns, food insecurity, or inadequate access to healthy food. Nutritional deficiencies, such as vitamin B12, B9 (folate), and zinc, can cause symptoms of depression such as low mood, fatigue, cognitive decline, and irritability [35,36]. In addition to specific nutrient deficiencies, patients with obesity have chronic inflammation. Evidence now strongly suggests the role of neuroinflammation in mental illness [37,38]. Inflammatory dietary patterns found in Western diets (i.e., processed food) are correlated with an increased risk of developing depression, mild cognitive impairment, and attention deficit hyperactivity disorder [39,40,41]. On the other hand, dietary patterns rich in whole foods, such as the Mediterranean diet, have been found to be protective against developing depression and other mental health symptoms [40,41,42].

Our findings should be considered within the context of limitations. A limited sample of postoperative patients completed the questionnaires at multiple time points. This is also confounded by the fact that attendance at multiple follow-up visits may be an indicator of greater overall well-being (e.g., including lower levels of anxiety and depression), compared to those who do not follow-up. The small sample size also exacerbated having a homogeneous sample, with all participants identifying as Caucasian and a majority identifying as female. It is unknown if results would differ with a more heterogeneous sample. Additionally, the prevalence of postoperative complications (e.g., gastrointestinal concerns, chronic pain, acid reflux, etc.) were not considered in this study, which could have impacted outcomes.

## 5. Conclusions

The current study evaluated the course of depression and anxiety symptoms after bariatric surgery. Results demonstrated that depression and anxiety symptoms decreased postoperatively and that anxiety symptoms reduced at a faster rate, comparatively, regardless of baseline BMI. The present results encourage additional research to examine anxiety and depression independently as outcomes following bariatric surgery. Since evaluating anxiety and depression symptoms as independent constructs following bariatric surgery has been uncommon, more research is needed to investigate the mechanisms for change. Understanding the contributing factors to quicker improvements in affective symptoms has strong implications for follow-up visits, building self-efficacy, and helping patients maintain sustainable healthy habits.

## Figures and Tables

**Table 1:** Demographic and descriptive statistics by time point.

Variable	Baseline (*n* = 27)	Time 1 (*n* = 18)	Time 2 (*n* = 18)	Time 3 (*n* = 12)
BMI	*n* = 26, 48.13 (7.16)	*n* = 18, 45.24 (6.61)	*n* = 18, 42.28 (6.23)	*n* = 12, 36.07 (3.92)
Change in BMI	--	*n* = 18, 4.14 (2.28)	*n* = 18, 4.78 (2.09)	*n* = 12, 9.86 (2.90)
PROMIS-D	*n* = 25, 12.52 (6.04)	*n* = 18, 10.67 (4.34)	*n* = 18, 11.33 (4.80)	*n* = 10, 9.80 (3.01)
PROMIS-A	*n* = 25, 13.24 (5.13)	*n* = 18, 10.78 (5.58)	*n* = 18, 10.89 (4.92)	*n* = 10, 11.40 (4.99)
Age	*n* = 27, 40.37 (9.92)	*n* = 18, 42.11 (9.78)	*n* = 18, 42.22 (9.35)	*n* = 12, 37.17 (9.31)
	N (valid %)	N (valid %)	N (valid %)	N (valid %)
Time since surgery				
Baseline	27 (100%)			
2 weeks		18 (100%)		
6 weeks			18 (100%)	
3 months				8 (66.7%)
6 months				4 (33.3%)
Birth Sex				
Female	24 (88.9%)	16 (88.9%)	15 (83.3%)	11 (91.7%)
Male	3 (11.1%)	2 (11.1%)	3 (16.7%)	1 (8.3%)
Race				
Caucasian	27 (100%)	18 (100%)	18 (100%)	12 (100%)
Marital Status				
Single/never married	7 (25.9%)	5 (27.8%)	4 (22.2%)	3 (25.0%)
Married	15 (55.6%)	9 (50.0%)	10 (55.6%)	8 (66.7%)
Divorced	3 (11.1%)	2 (11.1%)	3 (16.7%)	1 (8.3%)
Separated	1 (3.7%)	1 (5.6%)	1 (5.6%)	0 (0%)
Partnered	1 (3.7%)	1 (5.6%)	0 (0%)	0 (0%)
Education				
High school not completed	1 (3.7%)	0 (0%)	1 (5.6%)	1 (8.3%)
High school or GED	6 (22.2%)	5 (27.8%)	3 (16.7%)	0 (0%)
Some college	9 (33.3%)	4 (22.2%)	4 (22.2%)	6 (50.0%)
Associate degree	3 (11.1%)	3 (16.7%)	6 (33.3%)	0 (0.%)
Bachelor’s degree	3 (11.1%)	2 (11.1%)	1 (5.6%)	2 (16.7%)
Master’s degree or higher	5 (18.5%)	4 (22.2%)	3 (16.7%)	3 (25.0%)
Employment				
Full-time	24 (92.3%)	17 (94.4%)	15 (83.3%)	11 (91.7%)
Homemaker	1 (3.8%)	1 (5.6%)	1 (5.6%)	0 (0%)
Unemployed	1 (3.8%)	0 (0%)	2 (11.2%)	1 (8.3%)
Surgery Type				
Gastric sleeve	20 (76.9%)	14 (77.8%)	14 (77.8%)	9 (75.0%)
Gastric bypass	4 (15.4%)	2 (11.1%)	3 (16.7%)	3 (25.0%)
Revision from sleeve to bypass	2 (7.7%)	2 (11.1%)	1 (5.6%)	0 (0%)

Notes. BMI = body mass index. PROMIS-D = PROMIS Depression Short Form. PROMIS-A = PROMIS Anxiety Short Form. GED = general educational development.

**Table 2:** Generalized Estimating Equation with negative binomial distribution Incident Rate Ratio (IRR) results, main effect of time plus all covariates.

Outcome	Predictors	IRR	Lower 95% CI	Upper 95% CI	*p*-Value
BMI					
	Time 1	0.92	0.90	0.93	<0.0001
	Time 2	0.90	0.87	0.92	<0.0001
	Time 3	0.79	0.76	0.81	<0.0001
	Baseline	1.00	1.00	1.00	
	Education (>Highschool)	1.02	0.98	1.07	0.29
	Married	1.00	0.90	1.12	0.95
	Sleeve Surgery	0.99	0.95	1.03	0.58
	Age (yrs)	1.00	0.99	1.00	0.67
PROMIS-D					
	Time 1	0.83	0.67	1.02	0.08
	Time 2	0.92	0.73	1.15	0.44
	Time 3	0.78	0.62	1.00	0.05
	Baseline	1.00	1.00	1.00	
	Education (>Highschool)	0.93	0.75	1.15	0.48
	Married	0.77	0.65	0.92	0.003
	Sleeve Surgery	0.76	0.61	0.94	0.01
	Age (yrs)	0.99	0.98	1.00	0.01
	Baseline BMI	1.01	1.00	1.02	0.10
PROMIS-A					
	Time 1	0.80	0.64	1.01	0.07
	Time 2	0.81	0.67	0.99	0.04
	Time 3	0.85	0.62	1.16	0.30
	Baseline	1.00	1.00	1.00	
	Education (>Highschool)	1.01	0.76	1.34	0.95
	Married	0.80	0.65	0.98	0.03
	Sleeve Surgery	0.97	0.82	1.15	0.76
	Age (yrs)	0.99	0.98	1.00	0.03
	Baseline BMI	1.00	0.98	1.01	0.51

Notes. BMI = body mass index. PROMIS-D = PROMIS Depression Short Form. PROMIS-A = PROMIS Anxiety Short Form. Yrs = years.

**Table 3:** Results interpreted in the context of t-scores and minimally important differences.

	Raw Score	t-Score	∆ in t-Score	MID?
Depression				
Baseline	12.52	46.48		
Time 3	9.80	41.69	4.79	Yes
Anxiety				
Baseline	13.24	50.17	-	
Time 2	10.89	44.73	5.44	Yes

Notes. ∆ = change. MID = minimally important difference. Time 3 and Time 2 were selected for depression and anxiety, respectively, because these were the time points that a statistically significant difference was detected via GEE models.

## Data Availability

The data presented in this study are available on request from the corresponding author. The data are not publicly available due to respect the privacy of the participants.
